# Adenosine pathway regulates inflammation during *Plasmodium vivax* infection

**DOI:** 10.3389/fimmu.2023.1193256

**Published:** 2023-07-21

**Authors:** Suelen Queiroz Diniz, Maria Marta Figueiredo, Pedro Augusto Carvalho Costa, Olindo Assis Martins-Filho, Andrea Teixeira-Carvalho, Dhélio Batista Pereira, Mauro Shugiro Tada, Luis Carlos Crocco Afonso, Markus Kohlhoff, Carlos Leomar Zani, Ricardo Tostes Gazzinelli, Fabiano Oliveira, Lis Ribeiro Antonelli

**Affiliations:** ^1^ Laboratório de Biologia e Imunologia de Doenças Infecciosas e Parasitárias, Instituto René Rachou, Fundação Oswaldo Cruz, Belo Horizonte, Brazil; ^2^ Instituto de Ciências Biológicas, Departamento de Bioquímica e Imunologia, Universidade Federal de Minas Gerais, Belo Horizonte, Brazil; ^3^ Grupo Integrado de Pesquisas em Biomarcadores, Instituto René Rachou, Fundação Oswaldo Cruz, Belo Horizonte, Brazil; ^4^ Centro de Pesquisas em Medicina Tropical de Rondônia, Porto Velho, Brazil; ^5^ Instituto de Ciências Exatas e Biológicas, Departamento de Ciências Biológicas, Universidade Federal de Ouro Preto, Ouro Preto, Brazil; ^6^ Química de Produtos Naturais Bioativos, Instituto René Rachou, Fundação Oswaldo Cruz, Belo Horizonte, Brazil; ^7^ Laboratory of Malaria and Vector Research, National Institute of Allergy and Infectious Diseases (NIAID), National Institutes of Health, Rockville, MD, United States

**Keywords:** malaria, *Plasmodium vivax*, ectonucleotidases, adenosine, regulation

## Abstract

**Background:**

*Plasmodium* spp. infection triggers the production of inflammatory cytokines that are essential for parasite control, and conversely responsible for symptoms of malaria. Monocytes play a role in host defense against *Plasmodium vivax* infection and represent the main source of inflammatory cytokines and reactive oxygen species. The anti-inflammatory cytokine IL-10 is a key regulator preventing exacerbated inflammatory responses. Studies suggested that different clinical presentations of malaria are strongly associated with an imbalance in the production of inflammatory and anti-inflammatory cytokines.

**Methods:**

A convenience sampling of peripheral blood mononuclear cells from *Plasmodium vivax*-infected patients and healthy donors were tested for the characterization of cytokine and adenosine production and the expression of ectonucleotidases and purinergic receptors.

**Results:**

Here we show that despite a strong inflammatory response, monocytes also bear a modulatory role during malaria. High levels of IL-10 are produced during *P. vivax* infection and its production can be triggered in monocytes by *P. vivax*-infected reticulocytes. Monocytes express high levels of ectonucleotidases, indicating their important role in extracellular ATP modulation and consequently in adenosine production. Plasmatic levels of adenosine are not altered in patients experiencing acute malaria; however, their monocyte subsets displayed an increased expression of P1 purinergic receptors. In addition, adenosine decreases Tumor Necrosis Factor production by monocytes, which was partially abolished with the blockage of the A_2a_ receptor.

**Conclusion:**

Monocytes have a dual role, attempting to control both the *P. vivax* infection and the inflammatory response. Purinergic receptor modulators emerge as an untapped approach to ameliorate clinical malaria.

## 1 Introduction

Malaria is an infectious disease caused by protozoan parasites of the genus *Plasmodium.* Despite recent successes of several control actions implemented in endemic regions, malaria remains one of the greatest public health problems worldwide. *Plasmodium vivax* is the most widely distributed causative agent of human malaria, and is responsible for most cases in Latin America and Asia (1). Controlling the parasite burden without causing major host pathology benefits from a refined balance between inflammatory and regulatory immune responses, which failure is strongly associated with different clinical manifestations of the disease (2). Early production of pro-inflammatory cytokines, such as Tumor Necrosis Factor (TNF), Interleukin(IL)-6 and IL-1β, supports parasite clearance (3, 4) and, concomitantly, regulatory cytokines, such as IL-10, are also produced (2). IL-10 is a multifunctional cytokine with anti-inflammatory effect on most hematopoietic cell types, including monocytes and macrophages (5). A pre-clinical study reported that African children with severe anemia caused by *P. falciparum* display lower IL-10 levels than patients with moderate anemia, suggesting that IL-10 plays an important role in preventing severe cases (6). Moreover, the literature shows evidence that IL-10 may be protective by inhibiting TNF activity (7). Despite being considered a highly inflammatory disease, *P. vivax* infection triggers substantial IL-10 production (8) and although the immune response mediated by IL-10 during malaria is well established, the main sources, and what triggers its production are not fully understood.

In stress situations, such as cellular injury and infections, Adenosine Triphosphate (ATP) is released to the extracellular environment and act as a danger signal (9), binding to P2 purinergic receptors (10), stimulating robust inflammation mainly through the activation of monocytes, macrophages and dendritic cells (11). Therefore, extracellular ATP concentration is tightly regulated by the ectonucleotidases CD39 and CD73, which in cooperation hydrolyze ATP to adenosine (12). Adenosine binding to P1 purinergic receptors, A2a and A2b (13), triggers inhibition of pro-inflammatory cytokines (14) and stimulates the production of IL-10 (15). Considering these findings, we evaluated whether adenosine contributes to the regulation of *P. vivax* infection inducing inflammation.

We demonstrate that monocytes express high levels of ectonucleotidases, indicating their role in extracellular ATP modulation and, consequently, in adenosine production. Despite not detecting alterations on the plasmatic levels of adenosine in patients experiencing acute malaria caused by *P. vivax*, their monocytes displayed an increased expression of P1 purinergic receptors. In addition, adenosine decreases TNF production by monocytes, which is partially abolished with the blockage of the adenosine A_2a_ receptor.

## 2 Materials and methods

### 2.1 Population, patients, and healthy donors

A total of 48 patients with uncomplicated malaria caused by *P. vivax* infection were enrolled in this study at Centro de Pesquisa de Medicina Tropical de Rondônia (CEPEM) in Porto Velho, Rondônia, an endemic area for malaria in the Amazon region of Brazil. The group consisted of eight females (16.66%) and 40 males (83.66%) age range from 18 to 69 years (35 ± 11.13) (**Table S1**). Up to 100 mL of peripheral blood was collected after confirmation of *P. vivax* infection by thick blood smear film. Patients were treated according to the Brazilian Ministry of Health guideline. The clinical manifestations of acute malaria were fever, chills, nausea, vomit, diarrhea, myalgia, headache, and arthralgia. Peripheral blood was also collected from 11 healthy donors (HD) living in the same endemic area, and 20 individuals from Belo Horizonte/MG, nonendemic area for malaria. All the HD were tested for *P. vivax* infection and were negative.

### 2.2 Ethics statement

These studies were performed under protocols reviewed and approved by the Ethical Committees on Human Experimentation from Centro de Pesquisa em Medicina Tropical de Rondônia (CEP-CEPEM 095/2009) and Instituto René Rachou, Fundação Oswaldo Cruz (CEP-IRR 2004), the National Ethical Committee (CONEP 15652) from Ministry of Health, Brazil. Only adults, 18 years and older, were enrolled in the study. All patients enrolled in this study provided written informed consent.

### 2.3 Leukocyte purification

Peripheral blood mononuclear cells (PBMC) were prepared from heparinized venous blood of adult volunteers by Ficoll-Hypaque density gradient centrifugation (GE Healthcare Life Sciences). The blood samples were diluted in sterile 0.9% saline (vol/vol). Thirty-five microliters of the diluted blood was added gently into a tube containing 15 mL of Ficoll-Hypaque and centrifuged. PBMC were collected, transferred to a 50 mL conical tube, and washed three times. The cell concentration was determined using a Neubauer chamber and adjusted according to the test to be performed.

### 2.4 Reticulocyte purification and labeling

The red blood cell pellet from the Ficoll-Hypaque density gradient centrifugation was harvested and washed three times in Phosphate Buffered Saline (PBS) and then resuspended in RPMI 1640 medium (Sigma Aldrich) to a final hematocrit of 10%. Five milliliters of this suspension was overlaid on 5 mL of a 45% Percoll (Sigma Aldrich) solution in a 15 mL tube. After centrifugation, floating mature *P. vivax*-infected reticulocytes (*Pv*-RET) were collected, washed three times, and then labeled with FITC (200 µg/mL) (Sigma Aldrich). In brief, 10^6^ *Pv*-RET were resuspended in PBS and incubated for 30 minutes in the presence of Fluorescein-5-isothiocyanate (FITC) at room temperature, protected from light, and under constant agitation. Cells were washed twice and resuspended according to protocols to be used.

### 2.5 Plasmatic adenosine levels

The plasmatic adenosine levels were measured by targeted LC-MS analysis using a Nexera ultra-high performance liquid chromatography (UHPLC) system (Shimadzu, Japan) hyphenated to a maXis ETD high resolution ESI-qTOF mass spectrometer (Bruker, Germany) and controlled by the Bruker Compass v1.5 software Package. The peripheral blood (10 mL) was collected in a tube with heparin (12.4 U/mL) and a “stop solution” containing dipyridamole (75 μM), EHNA (15 μM), and ethylenediaminetetraacetic acid (3mM) were immediately added to the blood to prevent degradation/consumption of adenosine. After centrifugation (800 x g/10 min), the plasma was collected, and protein precipitation was performed using acetonitrile. Samples were centrifugated, and the supernatants were collected and stored at -80°C until analysis. A phenacetin solution (50 ng/mL) was prepared in acetonitrile (precipitation solution) and used as an internal standard to evaluate the efficiency of adenosine extraction. The separation of the compounds present in the supernatant was done using a Shimadzu Nexera UPLC coupled to a Bruker MaXis qTOF mass spectrometer. The column Shimadzu Shim-Pack XR-ODS III (C18, 2.2 μm, 80 A, and 2.0 x 200 mm) was used, and the amount of sample injected into the chromatograph was 10 μL. A binary gradient of mobile phase A (water) and B (acetonitrile) was programmed at a flow rate of 200 μL/min, both with 0.1% formic acid. In intervals of 0–0.5 min, 5% eluent B; 0.5–13 min, 5–100% B; 13–14 min, 100% B; 14–15 min, 100–5% B; and 15–20 min, 5% B were used. Throughout the process, the temperature was maintained at 40°C. Mass spectrometry was performed using the positive MS method with 0.5 Hz frequency and with a range of 40–400 m/z. A high precision calibration (HPC) was performed at the beginning of each run by injecting 20 μL of a 10 mM sodium formate/acetate solution.

### 2.6 Monocyte purification

CD14^+^CD16^−^, CD14^+^CD16^+^, and CD14^low^CD16^+^ monocytes from *P. vivax*-infected patients (Pv) were sorted with a FACSAria II cell sorter (BD Biosciences), using the following antibodies: anti-CD14 (clone 61D3)-BV450, anti-CD16 (clone 3G8)-PECy7, and anti-CD66b (clone G10F5)-FITC. Anti-CD66b was used to exclude contaminations with neutrophils.

### 2.7 mRNA detection

The analysis of the expression of messenger RNA (mRNA) was performed using the method of nCounter NanoString. A total of 10^4^ cells of each monocyte subpopulation were lysed in RLT buffer (QIAGEN) supplemented with β-mercaptoethanol and then frozen at -80°C. These lysates were hybridized with specific capture and reporter probes for 16 hours and loaded onto the nCounter prep station. The mRNA quantification was performed in the nCounter Digital Analyzer using 600 fields to detect the hybridized probes. Data were normalized in two ways described previously. Briefly, the first normalization was for small variations utilizing the internal positive controls present in each CodeSet. Then the samples were normalized with seven housekeeping genes that were included in the CodeSet. The data were analyzed with *n*Solver software.

### 2.8 Leukocyte immunophenotyping

Phenotypic characterization of peripheral blood leukocytes was performed using conventional and image flow cytometry. We evaluated the expression of the molecules of interest, CD39 (clone eBioA1 (A1))-PE CD73 (clone AD2)-PerCP-Cy5.5 in monocyte subsets, CD4^+^ and CD8^+^ cells, B cells and neutrophis. For these analyses, PBMC were incubated with surface antibodies (anti-CD14, anti-CD16, and anti-CD66b, anti-CD19 (clone HIB19)-FITC, anti-CD3 (clone OKT3)-FITC, anti-CD4 (clone SK3)-PE, anti-CD8 (clone RPA-T8)-eFluor780) and evaluated by conventional (BD FACSCelesta) and image flow cytometry (ImageStreamX Mark II, Merck-Millipore).

### 2.9 Colocalization assays

After purification of monocyte subsets by cell sorter, they were plated in Hanks’ Balanced Salt Solution (HBSS) medium and cultured for 3 hours in the absence (*Pv*-RET^-^) or in the presence (*Pv-*RET^+^) of *P. vivax*-infected reticulocytes labeled with FITC in a 1:1 ratio. After culture, the cells were washed and stained with surface antibodies (anti-CD39-PE and anti-CD73-PerCP-Cy5.5) for 30 minutes. After this period, the cells were rewashed, resuspended in 50 µL of staining buffer (PBS 2% fetal calf serum), and analyzed on the image flow cytometer (ImageStreamX Mark II, Merck-Millipore), which, in addition to generating quality images, allows statistical analysis. The analysis was performed using the IDEAS v6.1 software.

### 2.10 Cytokine production

Plasma levels of IL-10 were measured using the Th1/Th2/Th17 Cytometric Bead Array kit in plasma from Pv during acute disease following the manufacturer’s specifications. Intracellular IL-10 and TNF production by monocytes was assessed by conventional flow cytometry. Monocytes were stimulated with *Pv*-RET (1:1 ratio) or LPS (100 ng/mL) in the presence of brefeldin A (0.2 µL/200 µL) for 3 hours. After culture, cells were fixed, and permeabilized, and then incubated with antibodies anti-IL-10-PE and anti-TNF-FITC. The procedure was performed according to the manufacturer’s description (BD Cytofix/Cytoperm™).

### 2.11 Evaluation of adenosine modulation

To assess the ability of monocytes from Pv to modulate the immune response when stimulated by adenosine (10 nM), they were stimulated with lipopolysaccharides (LPS) (100 ng/mL) in the absence and the presence of adenosine. Subsequently, the intracellular levels of IL-10 and TNF were evaluated as described above. In specific experiments, ZM241385 (4-(2-(7-amino-2-(2-furyl)(1,2,4)triazole(2,3-α)(1,3,5) triazine-5-ylamino)ethyl)phenol) and MRS1754 (N-(4-cyanophenyl)-2(4-(2,3,6,7tetrahydro-2,6-dioxo-1,3-dipropyl-1H-purine-8yl)phenoxy)acetamide) (Tocris Bioscience), the ADORA_2a_ and A_2b_ antagonists respectively, were used. The cells were cultured for 30 minutes with the adenosine receptors antagonists (5 µM) before LPS (100 ng/mL) and adenosine (10 nM) were added.

### 2.12 Statistical analysis

The statistical analysis was performed, first considering the variables of independence, normality, and variance to define the data as parametric or nonparametric. Nonparametric data were evaluated using the Mann-Whitney test for unpaired samples or Wilcoxon matched-pair for paired samples. Parametric data were assessed by analysis of the unpaired or paired t-test. In all cases, the differences were considered statistically significant at *p* < 0.05. The GraphPad Prism 8.0 software was used for data analysis.

## 3 Results

### 3.1 IL-10 and ectonucleotidases CD39 and CD73 are induced during *P. vivax* infection

**Figure 1:**
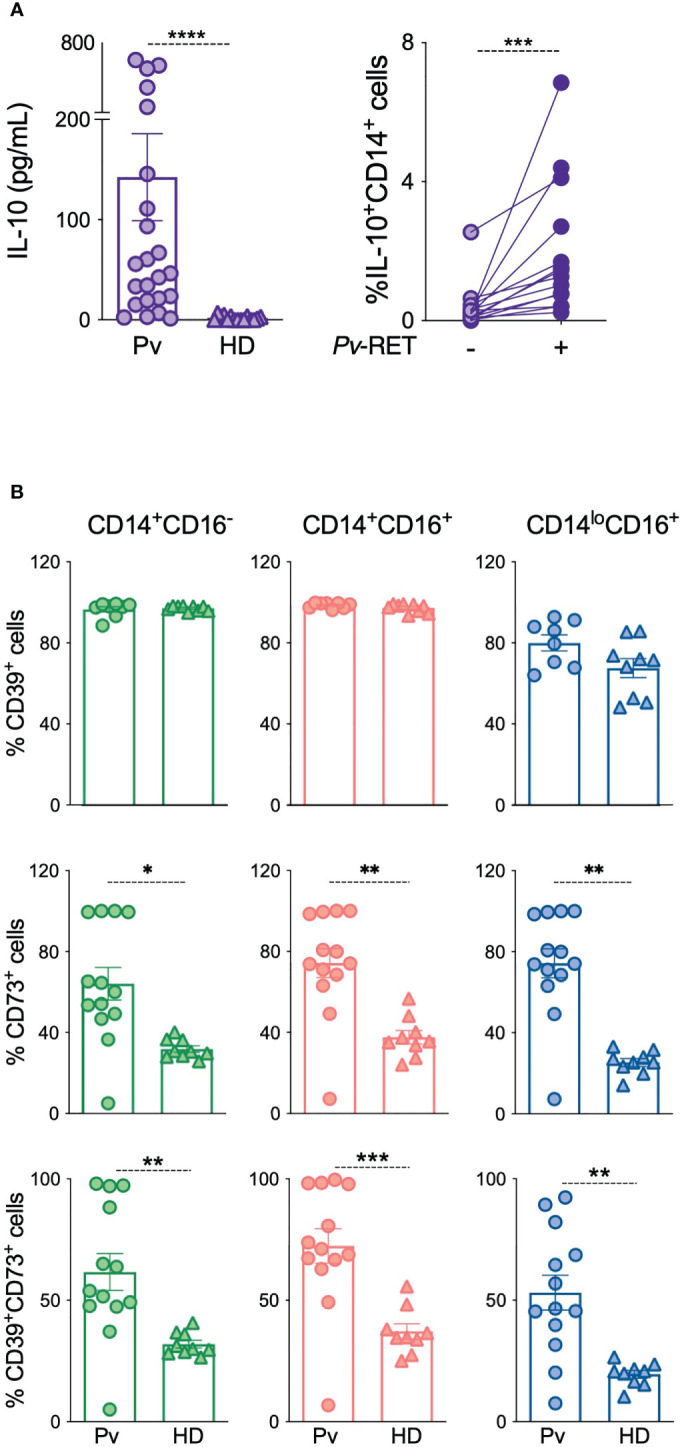
IL-10 and ectonucleotidases are upregulated in patients with acute *P. vivax* infection. **(A)** Plasma levels of IL-10 were measured using Th1/Th2/Th17 CBA kit in patients during acute malaria (Pv) (*n* = 23) and healthy donors (HD) (*n* = 13) (left panel). Intracellular IL-10 production by monocytes with (*Pv*-RET) and without (-) *P. vivax*-infected reticulocytes was assessed by flow cytometry (right panel) (*n* = 14) (right panel). **(B)** CD39, CD73 and their co-expression were determined in monocytes from Pv (*n* = 13) and HD (*n* = 9) using conventional flow cytometry in classical (green, left panel), inflammatory (red, middle panel) and patrolling monocytes (blue, right panel). Scatter plots with bars representing mean ± SEM. *p ≤ 0.05 **p ≤ 0.01 *** or ****p ≤ 0.001.

IL-10 levels were measured in the plasma from acute *P. vivax*-infected patients (Pv*)* as well as from HD (**Figure 1A**). Previous studies showed increased levels of circulating IL-10 during the acute phase of *P. vivax* infection (16). Likewise, we observe that Pv displayed statistically significant higher levels of IL-10 than HD (**Figure 1A**, left panel). IL-10 production was also induced by co-culturing monocytes with *Pv*-infected reticulocytes (*Pv*-RET) (**Figure 1A**, right panel).

Since IL-10 production can be triggered by adenosine, we investigated the expression of the ectonucleotidases CD39 and CD73, molecules responsible for extracellular adenosine production on circulating leukocytes from our cohort using flow cytometry (**Figure 1B** and **Figure S1**). No changes were detected on the CD39 expression in monocyte subsets from Pv compared to HD (**Figure 1B**, top panel), but its expression was increased on T cells during acute malaria (**Figure S1A**). Conversely, CD73 expression was higher on monocyte subsets from Pv compared to HD (**Figure 1B**, middle panel, but no changes were observed on T, B or neutrophils (**Figure S1B**). CD39 and CD73 are sequentially involved in the hydrolysis of extracellular ATP to adenosine, and their co-expression increases its efficiency. Therefore, we analyzed the co-expression of the ectonucleotidases, which followed the CD73 expression pattern, being induced during acute malaria (**Figure 1B**, bottom panel).

### 3.2 Inflammatory monocytes display higher levels of ectonucleotidases, which are not altered by *P. vivax*-infected reticulocytes

**Figure 2:**
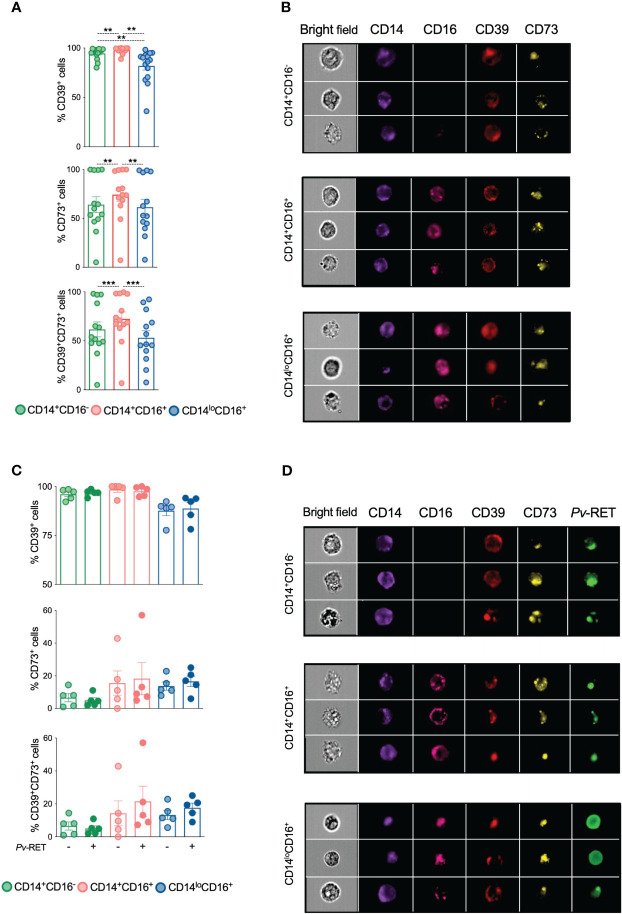
Ectonucleotidases CD39 and CD73 colocalize within monocyte subsets. CD39, CD73 and their co-expression were determined by imaging flow cytometry on monocyte subsets *ex vivo* (n = 13) **(A)** and after culture with (+) or without (-) *P. vivax*-infected reticulocytes **(C)** (*n* = 5). **p ≤ 0.01 ***p ≤ 0.001. Scatter plots with bars representing mean ± SEM. Green, red and blue symbols represent classical (CD14*^+^* CD16^-^), inflammatory (CD14^+^CD16^+^) and patrolling (CD14^lo^CD16^+^) monocytes **(A, C)**, respectively. Representative images of the expression of CD39 and CD73 **(B)** and *P. vivax*-infected reticulocytes and CD39 and CD73 **(D)** on monocyte subsets.

Image flow cytometry was used to define the levels of ectonucleotidases expression and their location on monocyte subsets. As shown by conventional flow cytometry, high frequencies of ectonucleotidases expressing cells were found among all monocyte subsets and were higher among inflammatory monocytes (**Figures 2A, B**, top and middle panels). Higher frequencies of CD39^+^CD73^+^ cells are also found in inflammatory monocytes from Pv than their other counterparts (**Figures 2A, B**, bottom panel, and **Figure S2A**).

We next asked if adding *P. vivax*-infected reticulocytes would alter the expression of ectonucleotidases on monocyte subpopulations *in vitro*. We observed that all three monocyte subsets were able to phagocytose *Pv*-infected reticulocytes (**Figures S2B, C**), which did not impact the expression of CD39 and CD73 (**Figures 2C, D** **)**.

### 3.3 *Plasmodium vivax* alters the transcription of P1 purinergic receptors genes in monocyte subpopulations

**Figure 3:**
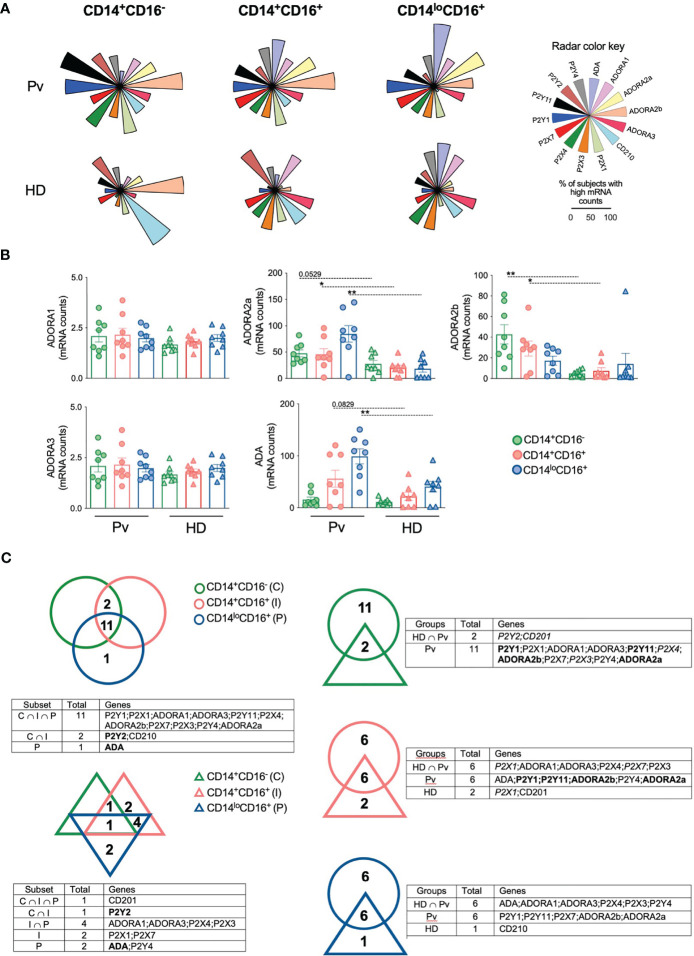
*Plasmodium vivax* infection alters the expression of adenosine and purinergic receptors in monocyte subsets. Gene expression was performed in monocyte subsets from HD (*n* = 8) and 8 Pv (*n* = 8) by NanoString analysis. **(A)** The proportion of subjects displaying high mRNA counts for each parameter was assessed using the overall median value as the cut-off, and radar graphs were assembled. Genes evaluated were ADA (purple); ADORA1 (pink); ADORA2a (yellow); ADORA2b (orange); ADORA3 (dark pink); CD210 (blue); P2X1 (green); P2X3 (dark orange); P2X4 (dark green); P2X7 (red); P2Y1 (dark blue); P2Y11 (black); P2Y2 (brown); and P2Y4 (gray). **(B)** Comparison of mRNA counts of adenosine receptors from monocyte subsets from Pv (circles) and HD (triangles). Scatter plots with bars representing mean ± SEM. *p ≤ 0.05 **p ≤ 0.01. **(C)** Venn diagrams of genes expressed in 50% or more subjects were built to identify common gene expression between each group. Gene expression among monocyte subsets from Pv (top, left panel) and HD (bottom, left panel). Genes in bold were commonly expressed by patients and HD in the same subset. Gene expression in each monocyte subset between Pv (circle) and HD (triangle) (right panel). Genes in bold were commonly expressed by Pv and HD or intersection group among the monocyte subsets analyzed. Genes in italic were exclusively expressed in a specific monocyte subset by Pv, HD or intersection group.

Extracellular ATP and adenosine act mainly through P2 and P1 purinergic receptor (10, 13), respectively. To determine whether *P. vivax* infection alter the expression of purinergic receptors, we analyzed the number of mRNA copies of selected genes in monocyte subsets from Pv and HD using nCounter NanoString. Fourteen genes were assessed: Adenosine deaminase (ADA) (purple), ADORA_1_ (pink), ADORA_2a_ (yellow), ADORA_2b_ (orange), ADORA_3_ (dark pink), IL-10 receptor (CD210) (blue), P2X_1_ (green), P2X_3_ (dark orange), P2X_4_ (dark green), P2X_7_ (red), P2Y_1_ (dark blue), P2Y_11_ (black), P2Y_2_ (brown), and P2Y_4_ (gray) (**Figure 3A**) (**Table S2**). The data obtained were organized in radar graphs, whose differences in shapes indicate that *P. vivax* infection changes the expression of purinergic receptors (**Figure 3A**). The expression of ADORA_2a_ and ADORA_2b_ were increased respectively in inflammatory and patrolling monocytes and classical and inflammatory monocytes from Pv compared with HD (**Figure 3B**). *P. vivax* infection also increased the expression of ADA in patrolling monocytes. Higher levels of ADORA_2b_ are found in classical than in patrolling monocytes and higher levels of ADA are found in the patrolling subset followed by their inflammatory and classical counterparts. The significant changes observed in the expression of ectonucleotidases and the adenosine receptors ADORA_2a_ and ADORA_2b_ in monocyte subsets, suggest a role for adenosine in malaria. Fewer significant differences were found in P2 purinergic receptors (**Table S2**).

Venn diagrams were built to investigate the similarities and differences among monocyte subsets within the same context. Monocyte subsets from HD expressed only 10 genes in 50% or more subjects, and only CD210 was highly expressed in all subsets (**Figure 3C**, left lower panel). Noteworthy, 11 genes were expressed by the three subsets from Pv (**Figure 3C**, left top panel). When each monocyte subset from Pv and HD was compared, we observed that despite expressing a considerable number of genes in common, most of the genes analyzed are highly expressed only in the monocytes from Pv (**Figure 3C**, right panel), corroborating the hypothesis that Pv infection causes substantial changes in the expression of purinergic receptors by monocyte.

To better explore the impact of malaria on the expression of purinergic and IL-10 receptors, the proportion of subjects displaying each gene was represented as an ascending signature built using each gene’s overall median value (**Figure S3**). Those parameters observed in 50% or more of the subjects analyzed were highlighted for each monocyte subset. At least 50% of Pv express most of the genes analyzed above the overall median, but higher frequencies of lower expressors are observed in HD (**Figure S3**). Almost all genes analyzed were highly expressed in monocyte subsets among Pv, except for ADA in classical monocytes; and CD210 and P2Y_2_ in inflammatory and patrolling monocytes.

### 3.4 Adenosine decreases TNF production by monocytes in an ADORA_2a_ dependent manner

**Figure 4:**
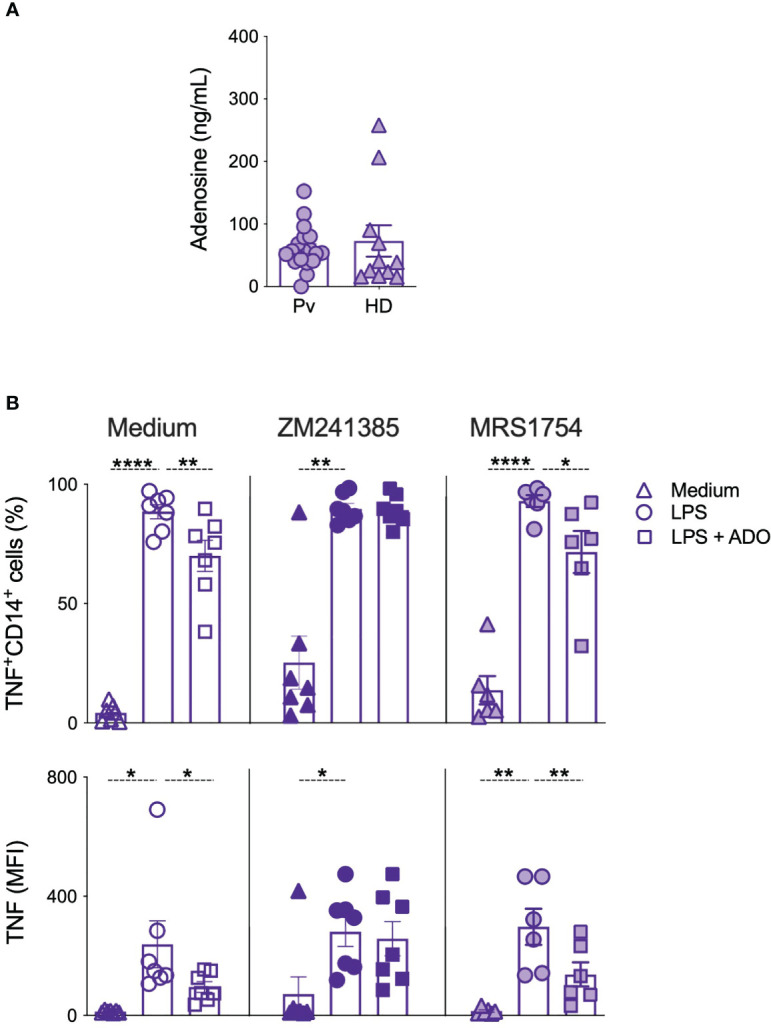
Adenosine decreases TNF production by monocytes in an adenosine 2a receptor depend-manner. **(A)** Adenosine levels were measured in the plasma of Pv (*n* = 30) and HD (*n* = 11) by UHPLC. **(B)** Frequencies (top panels) and mean fluorescence intensity (MFI) of TNF-producing monocytes were measured after culture with medium alone (triangles), LPS (circles), and LPS + adenosine (squares) and in the absence (open symbols) or presence of adenosine 2a receptor blocker (ZM241385, solid symbols) or adenosine 2b receptor blocker (MRS1754, gradient symbols) (n = 6–7). Scatter plots with bars representing mean ± SEM. *p ≤ 0.05 **p ≤ 0.01 ****p ≤ 0.001.

The significant changes observed in the expression of ectonucleotidases, and purinergic receptors suggest a role for adenosine during *P. vivax* infection. Systemic levels of adenosine, measured in the plasma, from Pv and HD (**Figure 4A**) were similar suggesting that their main role is in the cell microenvironment (**Figure 4A**). When monocytes from Pv were stimulated with LPS we observed a reduction on IL-10 levels that was not rescued by adenosine (**Figure S4**). However, adenosine triggered a decrease in the frequencies of TNF-producing monocytes from Pv stimulated with LPS (**Figure 4B**). In addition, the reduction of TNF production by monocytes induced by adenosine was reverted using ZM241385, a blocker for ADORA_2a_. The same was not observed with MRS1754, an ADORA_2b_ antagonist (**Figure 4B**). These results indicate that adenosine act through ADORA_2a_ diminishing the production of the inflammatory cytokine TNF by monocytes.

## 4 Discussion

*Plasmodium* spp. triggers the production of high levels of inflammatory cytokines and their signaling are essential for both parasite control and symptoms observed during acute disease (2). Induction of IL-10 production is frequently observed in *Pv*-infected patients and previous studies have suggested a role for this cytokine in controlling immunopathology in human malaria. Individuals infected with *P. vivax* produce higher levels of circulating IL-10 than patients infected with *P. falciparum* (8), and different clinical manifestations of the disease are strongly associated with an imbalance in the production of inflammatory and regulatory cytokines (17).

Adenosine is a regulatory molecule associated with the control of the inflammatory responses by stimulating IL-10 production (15). Adenosine measurements in blood/plasma are technically difficult and unreliable due to its short life and rapid clearance (18). No adenosine systemic levels have been reported in malaria, and we detected no measurable increases in the adenosine levels during *P. vivax* infection. Nevertheless, measuring the expression of ectonucleotidases and adenosine receptors can be used as a substitute for direct measurements of adenosine levels.

The expression of ectonucleotidases is upregulated by pro-inflammatory cytokines, oxidative stress and hypoxia (19), conditions commonly observed during malaria. Previous studies showed that CD39 is heterogeneously expressed in leukocytes (20), being expressed in approximately 90% of neutrophils, monocytes, and B cells. Our results confirm these findings in both *Pv*-infected and healthy individuals. Classical and inflammatory monocytes expressed higher levels of CD39, whose expression is not altered by *P. vivax* infection. However, higher frequencies of CD73 and CD39/CD73 expressing cells were found among monocyte subsets from *Pv*-infected compared to HD. Taken together, CD73 overexpression in monocytes during *P. vivax* malaria may contribute to adenosine production. Moreover, since CD39 and CD73 correspond to sequential steps of hydrolysis of ATP to adenosine, it is plausible to think that their co-expression increases their efficiency. Our group demonstrated monocytes were also able to produce IL-10 when stimulated with *Pv*-RET (16). Interestingly, the *Pv*-RET phagocytosis did not alter the expression of ectonucleotidases in monocyte subsets, suggesting that the induction in the CD73 expression in monocytes from *P. vivax*-infected patients was due to the environment caused by the *P. vivax* infection.

Adenosine signals through surface P1 purinergic receptors expressed by immune cells (10, 13). We demonstrated that during acute *P*. *vivax* infection, monocyte subsets displayed distinct pattern of expression of P1 receptors. The role of P1 purinergic receptors has been extensively associated with the function of cells from the immune system (21), whereas TNF-induced A_2a_ receptor as a negative feedback control (22). The binding of adenosine to A_2a_ and A_2b_ receptors inhibited TNF production (23) and increased IL-10 (15) release by macrophages. Accordingly, it has been shown that A_1_ receptors activation on monocytes enhanced Fcγ receptor-mediated phagocytosis, whereas A_2_ receptors reduced phagocytosis in the same cells (24). In addition, A_2b_ activation also stimulated IL-10 and inhibited IL-12 and LPS-induced IL-18 production by human monocytes (15, 25). *P. vivax* infection increased the expression of A_2a_ and A_2b_ receptors in monocyte subsets, supporting the role for the adenosine system in immunomodulation during malaria. Adenosine inhibits LPS-induced TNF production in mouse macrophage cell lines (26). Our results confirmed these findings in human monocytes from Pv-infected individuals and that CD14^+^TNF^+^ cell frequency is reconstituted by blocking A_2a_ receptor signaling by an antagonist. Notably, the same was not observed by using the A_2b_ antagonist. Previous studies have shown that a high concentration of adenosine is necessary to induce IL-10 compared to the concentration capable of inhibiting TNF production (27). This might be one of the reasons we were not able to detect the modulation of IL-10 production by A_2a_ receptor signaling.

Our work raise several questions that we hope to address in the future, such as the potential role of adenosine pathways in different clinical presentations of malaria, including correlation with the subject parasitemia, number of acute malaria episodes and even in asymptomatic Pv infections. Also it remains to be identified the mechanistic nature by which ectonucleotidases expression is modulated by the environment or the systemic alterations caused by Pv infection.

Altogether, our findings demonstrated that monocyte subsets expressed high levels of the ectonucleotidases CD39 and CD73, suggesting a role for these cells in adenosine production and consequently in the modulation of inflammatory response stimulated by malaria parasites. Despite not detecting differences in plasmatic circulating adenosine levels, we demonstrate that monocyte subsets from *Pv*-infected patients displayed augmented expression of A_2a_ and A_2b_ receptors, indicating a potential increase in the consumption of adenosine. In addition, we demonstrated that adenosine modulates inflammatory response by decreasing TNF production by monocytes, which is restored with the blockage of ADORA_2a_. Ultimately, the present study attempted to contribute to the knowledge of immunoregulation during malaria.

## Data availability statement

The raw data supporting the conclusions of this article will be made available by the authors, without undue reservation.

## Ethics statement

The studies involving human participants were reviewed and approved by Committees on Human Experimentation from Centro de Pesquisa em Medicina Tropical de Rondônia (CEP-CEPEM 095/2009) and Instituto René Rachou, Fundação Oswaldo Cruz (CEP-IRR 2004), the National Ethical Committee (CONEP 15652) from Ministry of Health, Brazil. The patients/participants provided their written informed consent to participate in this study.

## Author contributions

SD, LVA designed the research. SD, AC, MF, PC, MK, CZ performed experiments and discussed data. RG, LCA discussed data and provided reagents. DP, MT participated in the enrollment and provided clinical care. SD, OM-F, RG, FO, LVA performed data analysis. SQD, FO, LA wrote the manuscript. All authors contributed to the article and approved the submitted version.
