# Supplementary material for: Adenosine pathway regulates inflammation during Plasmodium vivax infection
Source: Front Immunol. 2023 Jul 21;14:1193256. doi: 10.3389/fimmu.2023.1193256 (PMC10402272; doi:10.3389/fimmu.2023.1193256)
Supplement: Supplementary file 1 [file DataSheet_1.pdf]

Supplementary materials

Figure S1

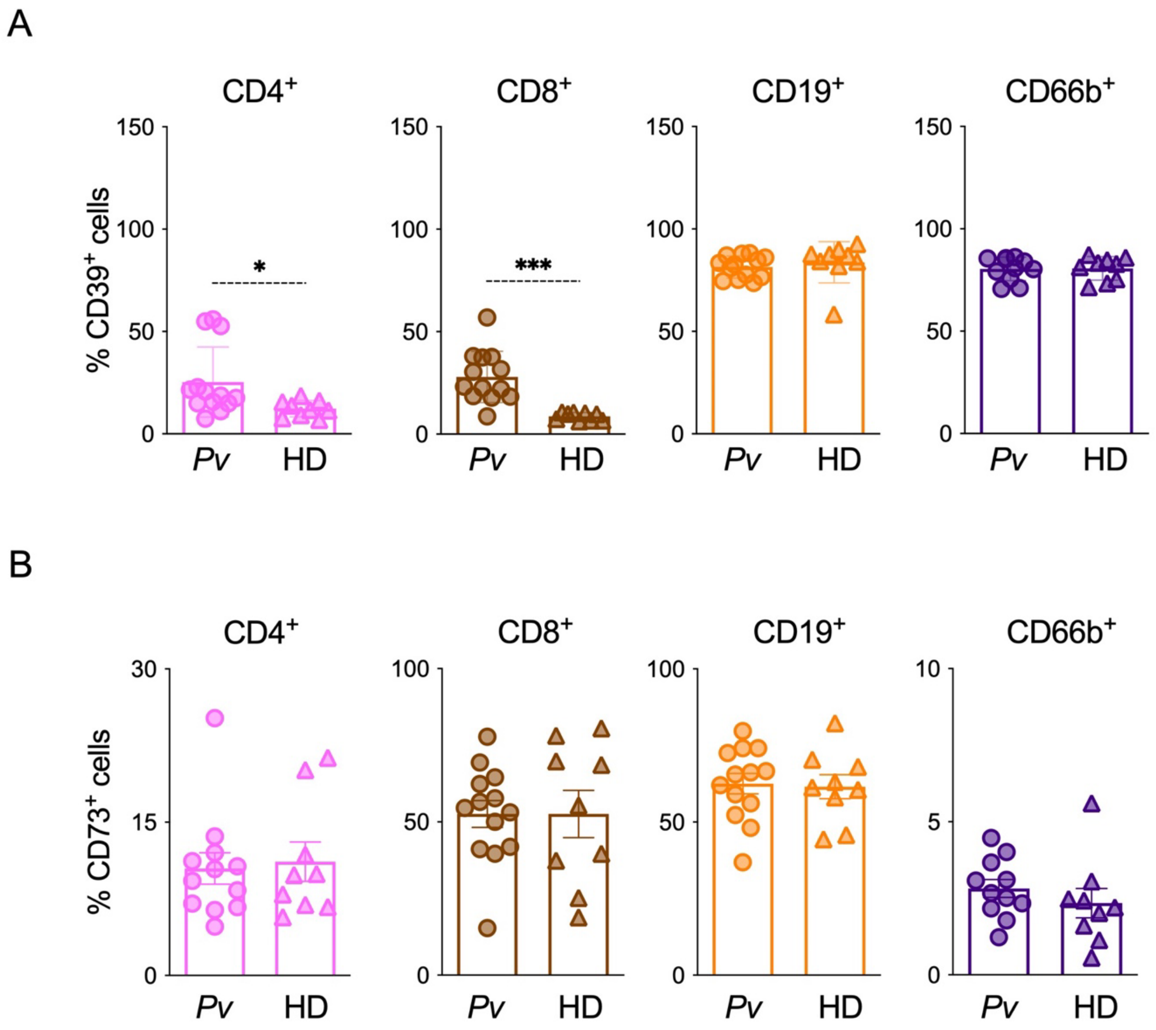

Figure S1. CD39 is upregulated in T cells from patients with acute *P. vivax* infection. CD39 (A) and CD73 (B) were determined in $CD4^+$ and $CD8^+$, B cells ($CD19^+$) and neutrophils ($CD66^+$) from Pv (*n* = 13) and HD (*n* = 9) using conventional flow cytometry. Scatter plots with bars representing mean ± SEM. * $p \leq 0.05$ *** $p \leq 0.001$.

Figure S2

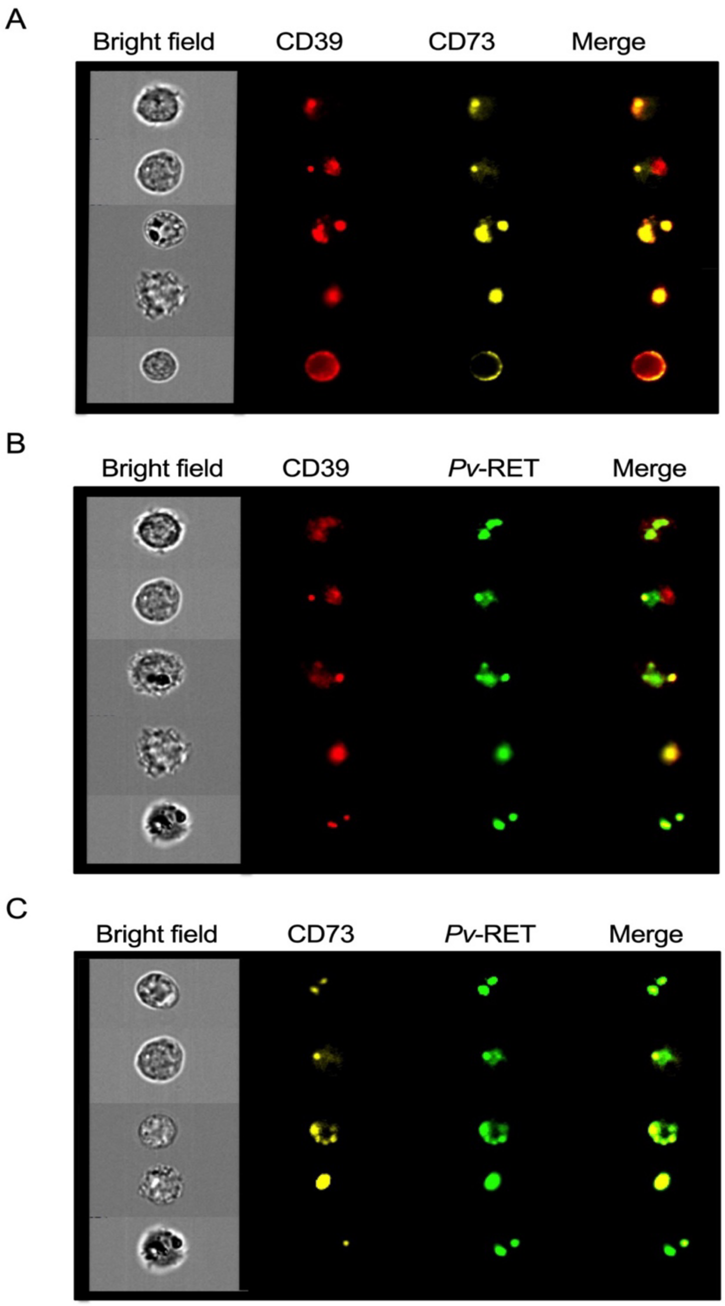

Figure S2. Co-expression of ectonucleotidases and *P. vivax*-infected reticulocytes. Representative images of flow cytometry of the co-expression of CD39 and CD73 (A), and *P. vivax*-infected reticulocytes and CD39 (B) and CD73 (C) on monocytes.

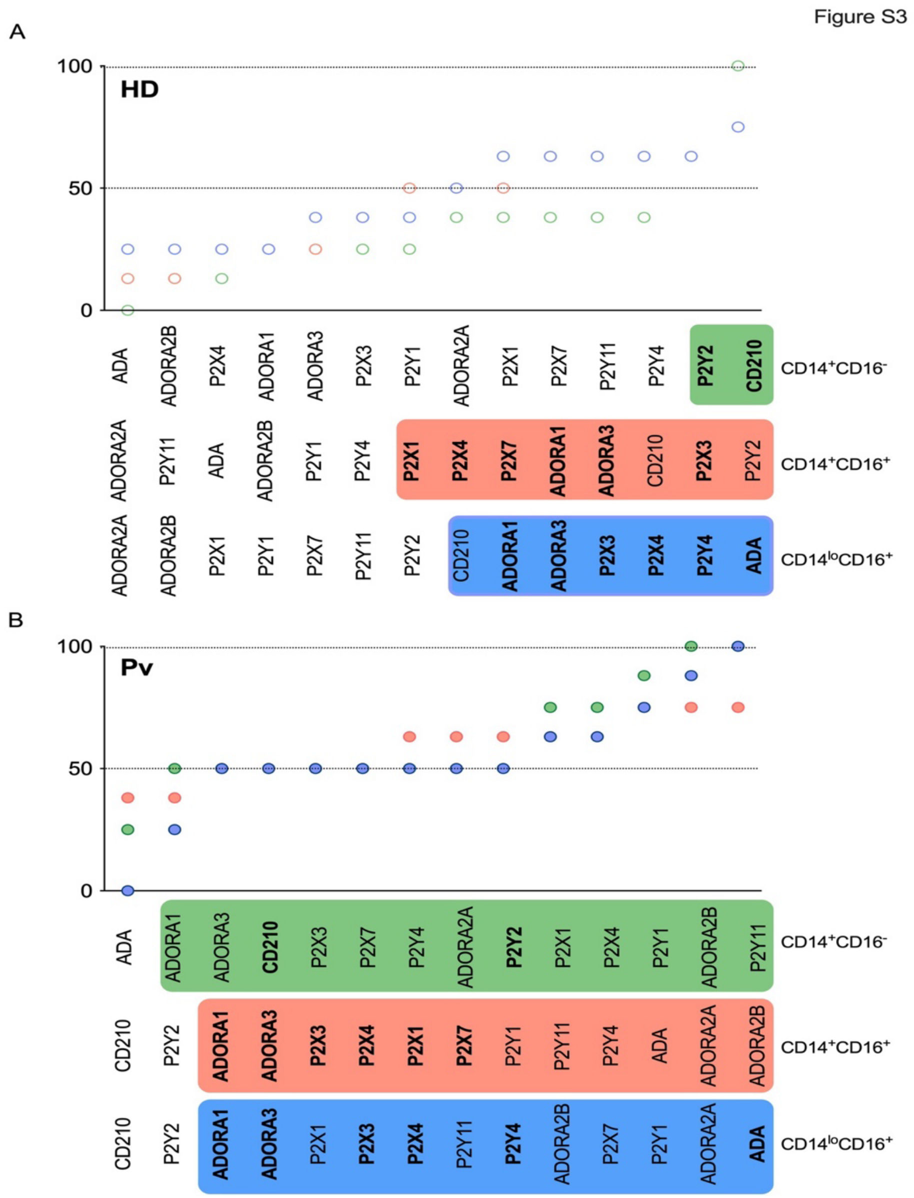

Figure S3. *P. vivax* infection alters ascendant signatures in monocyte subsets. The proportion of subjects displaying each parameter was assessed using the overall median value, and ascendant signatures were assembled. Those parameters observed in 50% or more subjects were highlighted for each subset from HD (A) and Pv (B). Classical monocytes were represented in green, inflammatory monocytes in red, and patrolling monocytes in blue. Genes in bold represent the parameters observed in 50% or more subjects in the same monocyte subset from Pv and HD.

Figure S4

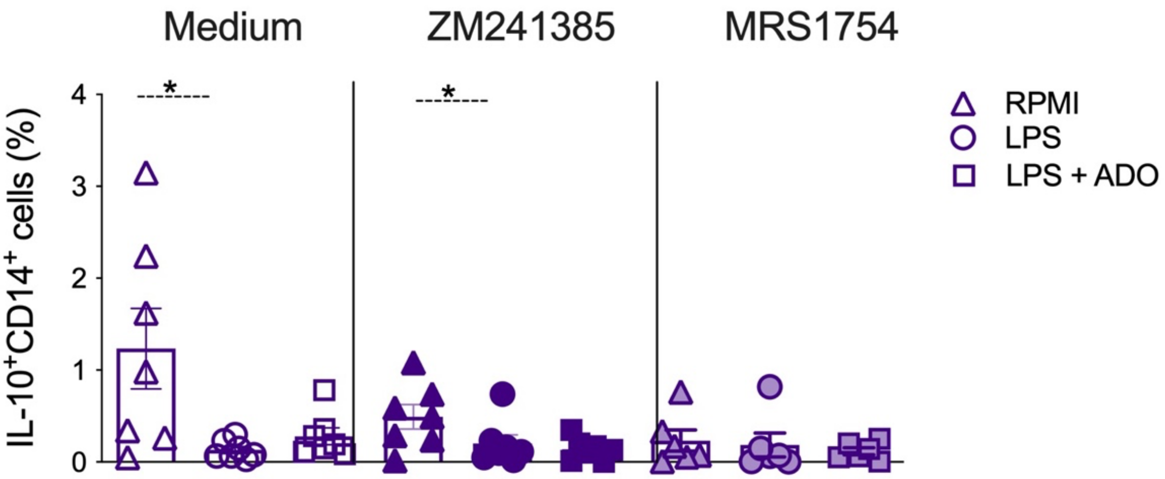

Figure S4. IL-10 production by monocytes from *P. vivax* patients. Frequencies of IL-10-producing monocytes were measured after culture with medium alone (triangles), LPS (circles), and LPS + adenosine (squares) and in the absence (open symbols) or presence of adenosine 2a receptor blocker (ZM241385, solid symbols) or adenosine 2b receptor blocker (MRS1754, gradient symbols) (n = 6–7). Scatter plots with bars representing mean ± SEM. * $p \leq 0.05$.

Table S1 - Anamnesis and Clinical Examination. Gender, age, number of previous malaria episodes, parasite load, and symptoms of *P. vivax*-infected patients.

Table S1 - Anamnesis and Clinical

Gender (male, %)	81.25
Age (mean ± SD)	35.56 ± 11.30
Malaria episodes	%
1st	34.09
< 5	36.36
≥ 5	29.54
Parasite/μL	%
≤ 500	18.60
500–10,000	72.09
> 10,000	09.30
Symptoms	%
Fever	81.25
Chills	72.91
Nausea	45.83
Vomit	14.58
Diarrhea	06.25
Myalgia	91.66
Headache	85.41
Arthralgia	72.91
Jaundice	0.00

Table S2 - Statistical differences between mRNA count in monocyte subsets from healthy donors and *P. vivax*-infected patients. Genes were divided into three groups based on their role in adenosine: ADA, ADORA1, ADORA2a, ADORA2b, ADORA3 (blue); IL-10: CD210 (yellow) or ATP metabolism: P2X1, P2X3, P2X4, P2X7, P2Y1, P2Y11, P2Y2, and P2Y4 (pink).

Table S2 – Statistical differences between mRNA counts in monocyte subsets from healthy donors and *P. vivax*-infected patients

Official full name	Gene ID	*p* values		
		$CD14^+CD16^-$	$CD14^+CD16^+$	$CD14^{lo}CD16^+$
Adenosine deaminase	ADA	0.396	0.082	**0.005**
Adenosine A1 receptor	ADORA1	0.215	0.349	0.972
Adenosine A2a receptor	ADORA2A	**0.052**	0.044	**0.001**
Adenosine A2b receptor	ADORA2B	**0.003**	**0.016**	0.780
Adenosine A3 receptor	ADORA3	0.215	0.349	0.972
Interleukin 10 receptor subunit alpha	CD210	**0.003**	0.062	0.393
Purinergic receptor P2X1	P2X1	0.093	0.584	0.639
Purinergic receptor P2X3	P2X3	0.215	0.349	0.972
Purinergic receptor P2X4	P2X4	0.123	0.802	0.220
Purinergic receptor P2X7	P2X7	0.670	0.694	0.280
Purinergic receptor P2Y1	P2Y1	**0.022**	0.854	**0.056**
Purinergic receptor P2Y11	P2Y11	**0.006**	0.291	0,569
Purinergic receptor P2Y2	P2Y2	0.194	0.092	0.347
Purinergic receptor P2Y4	P2Y4	0.300	0.252	0.972

Genes were divided into three groups based on their role in adenosine (blue), IL-10 (yellow), or ATP (pink) metabolism. HD *n* = 11, Pv *n* = 13.
